# Survival Prediction of Patients after Heart Attack and Breast Cancer Surgery with a Hybrid Model Built with Particle Swarm Optimization, Stacked AutoEncoders, and the Softmax Classifier

**DOI:** 10.3390/biomimetics9050304

**Published:** 2024-05-19

**Authors:** Mehmet Akif Bülbül, Mehmet Fatih Işık

**Affiliations:** 1 Department of Software Engineering, Kayseri University, Kayseri 38280, Turkey; 2 Department of Electrical-Electronics Engineering, Hitit University, Çorum 19030, Turkey; mehmetfatih@hitit.edu.tr

**Keywords:** decision support system, stacked autoencoder, particle swarm optimization, softmax classifier, survival after breast cancer surgery, survival after heart attack, decision support system

## Abstract

The prediction of patient survival is crucial for guiding the treatment process in healthcare. Healthcare professionals rely on analyzing patients’ clinical characteristics and findings to determine treatment plans, making accurate predictions essential for efficient resource utilization and optimal patient support during recovery. In this study, a hybrid architecture combining Stacked AutoEncoders, Particle Swarm Optimization, and the Softmax Classifier was developed for predicting patient survival. The architecture was evaluated using the Haberman’s Survival dataset and the Echocardiogram dataset from UCI. The results were compared with several Machine Learning methods, including Decision Trees, K-Nearest Neighbors, Support Vector Machines, Neural Networks, Gradient Boosting, and Gradient Bagging applied to the same datasets. The findings indicate that the proposed architecture outperforms other Machine Learning methods in predicting patient survival for both datasets and surpasses the results reported in the literature for the Haberman’s Survival dataset. In the light of the findings obtained, the models obtained with the proposed architecture can be used as a decision support system in determining patient care and applied methods.

## 1. Introduction

Health is paramount in people’s lives, representing the most invaluable aspect. However, health-related adversities often necessitate extensive treatment processes. Particularly, diseases requiring prolonged treatment or exhibiting a low probability of recovery can impose significant challenges on individuals. Such challenges underscore the critical importance of early detection and effective management, particularly for diseases amenable to treatment. Surgery, in particular, marks a pivotal juncture in disease management [1]. Predicting survival is an important parameter for understanding patient treatment and recovery. Predictions are essential to help healthcare providers develop treatment plans and evaluate treatment outcomes. Accurate prediction is crucial for developing the most appropriate treatment plan and managing the lives of patients [2].

In this study, our primary objectives were twofold. Firstly, we aimed to enhance the accuracy of predicting survival duration for patients diagnosed with breast cancer and those who have experienced heart attacks. This would be achieved through the integration of Stacked AutoEncoders, the Particle Swarm Optimization algorithm, and the Softmax Classifier. Stacked AutoEncoders are one of the most popular models in the field of Deep Learning. The Particle Swarm Optimization algorithm is frequently used in the literature and is an effective optimization algorithm in solving multidimensional problems. The Softmax Classifier is a classification method that attracts attention with its high classification performance. By harnessing the strengths of these methodologies, our goals was to provide more precise prognostications, ultimately improving patient care and treatment outcomes. Secondly, we endeavored to develop a methodology capable of generating diverse and effective architectures tailored to specific problem domains. This would involve crafting adaptable frameworks that could accommodate various datasets and challenges inherent to different medical scenarios. By designing such a methodology, we aimed to contribute to the broader field of Machine Learning by providing a flexible and scalable approach to solving complex problems in healthcare and beyond.

In alignment with these aims and objectives, a Deep Learning Network was constructed, leveraging an optimization approach that integrated Stacked AutoEncoders, the Particle Swarm Optimization algorithm, and the Softmax Classifier. This amalgamation aimed to enhance the network’s performance in predicting survival duration for patients diagnosed with breast cancer and those affected by heart attacks. To tailor the network to the specific tasks at hand, both the architectural parameters and the hyperparameters were meticulously optimized using the Particle Swarm Optimization algorithm. This optimization process aimed to fine-tune the network’s configuration for optimal performance, ensuring its efficacy in addressing the targeted medical prognostication tasks. The developed architecture was then put to the test using real-world datasets obtained from the Haberman’s Survival and Echocardiogram datasets sourced from the UCI repository. Additionally, various conventional Machine Learning methods were employed on these datasets to provide a basis for comparative analysis. The results derived from the hybrid architecture were juxtaposed with outcomes from alternative Machine Learning methods and existing studies within the literature that utilized identical datasets. This comparison was conducted to provide a comprehensive evaluation, and the findings are presented in a comparative manner.

Innovations derived from the experimental studies: The proposed hybrid architecture demonstrated superior success rates compared to the Machine Learning methods employed in the research. Furthermore, it achieved notably higher success rates on the Haberman’s Survival dataset compared to those reported in the existing literature. This study introduces a novel hybrid Machine Learning approach that integrates Stacked AutoEncoders, the Softmax Classifier, and Particle Swarm Optimization. Additionally, an alternative decision support system is proposed for predicting the survival outcomes of patients following breast cancer surgery and heart attack incidents.

## 2. Literature Review

There are many studies in the literature for patient survival predictions. For example, Li and Wang [3] performed survival probability estimation with the semi-parametric model averaging method. In the study, a Brier Score-type criterion was used to select the most appropriate model, which was the average of the weights. In the study, successful results were obtained in predicting the survival probabilities of patients with heart failure.

Evangeline et al. [4] used three different models—namely, the Cox Proportional Hazards (CoxPH) model, the Random Survival Forests (RSF) model, and DeepHit—in their study for the survival prediction of breast cancer patients. As a result of these experimental studies, successful results were obtained in the RFS and DeepHit models.

Hussain et al. [5] proposed a model to predict the survival time of patients with brain tumors. This model was the first model to consider the volume of brain tumor in survival time calculation. In the study, where 4D-MRI, 3D CNN, and CoxPH models were used, it was emphasized that more successful results were obtained than the leading survival prediction models.

Huang et al. [6] used Machine Learning and Deep Learning techniques to predict the survival times of patients with lung cancer. Using 12 demographic and clinical characteristics, the study applied seven Machine Learning models, including Logistic Regression (LR), Bayesian Classifier (BayesNet), Lazy Classifier (LWL), Meta-Classifier (AttributeSelectedClassifier (ASC)), Rule Learner (OneR), Decision Tree (J48), and Deep Neural Network (DNN). Among the models used, DNN was more successful than the other methods.

Atallah et al. [7] proposed a hybrid model to predict survival in the next five years after kidney transplantation. In the hybridization of the pure Bayes and K-Nearest Neighbor Machine Learning methods, successful results were obtained in predicting survival after kidney transplantation.

Azam et al. [8] presented a novel approach aimed at enhancing accuracy in classification processes. This approach focused on generating Type-1 Fuzzy Triangular and Trapezoidal membership functions. The study employed the generation of c-means clustering, membership matrix, and cluster centers to produce Type-1 Fuzzy membership functions. It has been applied to various datasets, including the Haberman’s Survival dataset. The findings from the experimental study indicate the success of the approach.

Kurama [9] proposed a novel similarity-based classifier integrated with aggregator operators. Four distinct Dombi aggregation operators—conjunctive, discrete, weighted conjunctive, and product operators—were employed to aggregate similarities within the classifier. Utilizing these proposed methods, the accuracy rate for predicting patient survival after surgery increased by 0.29 compared to the existing literature.

Bataineh et al. [10] utilized the Clonal Selection Algorithm for multi-layer sensor training in their work. The study also employed Genetic Algorithms, Ant Colony Optimization, Particle Swarm Optimization, Harris Hawks Optimization, Moth Flame Optimization, Flower Pollination, and Backpropagation methods to compare the performance of the proposed method. Successful results were achieved with the proposed methods in the study, where all the methods were used to estimate patient survival after surgery.

Kaushik et al. [11] have proposed a new approach for predicting the survival of breast cancer patients. The new approach, recommended for lumpectomy and mastectomy, utilizes Support Vector Machine Communication-Efficient Distributed Double Coordinate Ascent (SVM-CoCoA). The originality of the new model was observed through an experiment conducted using the Simple Compilation Tool (SBT) and Apache Flink. Experimental studies were carried out using the Haberman’s Survival dataset. The findings revealed the success of the proposed method.

Aljawad et al. [12] used Bayes Network and Support Vector Machines to estimate the survival status of patients undergoing breast cancer surgery. The results from experimental studies conducted on the Weka software platform showed that the Support Vector Machines method produced more successful results than the Bayes Network.

Remya Ajai et al. [13] conducted an analysis of logistically mapped neurons in neuroacoustic learning architectures for data classification. The study proposed two different extensions of Neurochaos Learning, a chaos-based learning algorithm for classification. To further enhance the classification performance of 1D logistic map neurons and the Heterogeneous Neuroacoustic Learning Architecture, Chaos-based attributes were extracted from the Extruded and Support Vector Machines instead of the GLS Neurons. Successful results were obtained in the approaches used to predict the survival of patients undergoing breast cancer surgery.

The utilization of Machine Learning (ML) methods in health decision support systems is witnessing a steady rise. This trend holds significant importance for healthcare professionals in discerning the most appropriate treatment methods and assessing the efficacy of applied treatments. ML methods employed in the healthcare domain encompass numerous parameters, making it exceedingly challenging to fine-tune these parameters specifically for a given problem through trial-and-error methods alone. In this context, integrating various ML methods and optimizing the hyperparameters within established architectures play a pivotal role in achieving successful outcomes. In light of all this, in this study, a hybrid model is proposed that creates the most suitable model for the problems by optimizing its own architectural structure and the hyperparameters within the architecture.

## 3. Materials and Methods

In this section, the Particle Swarm Optimization Algorithm used in the study, Stacked AutoEncoders, the Softmax Classifier, the other ML methods used, and the metrics used to evaluate the performance of the proposed hybrid architecture will be explained in detail.

### 3.1. Particle Swarm Optimization Algorithm

**Figure 1:**
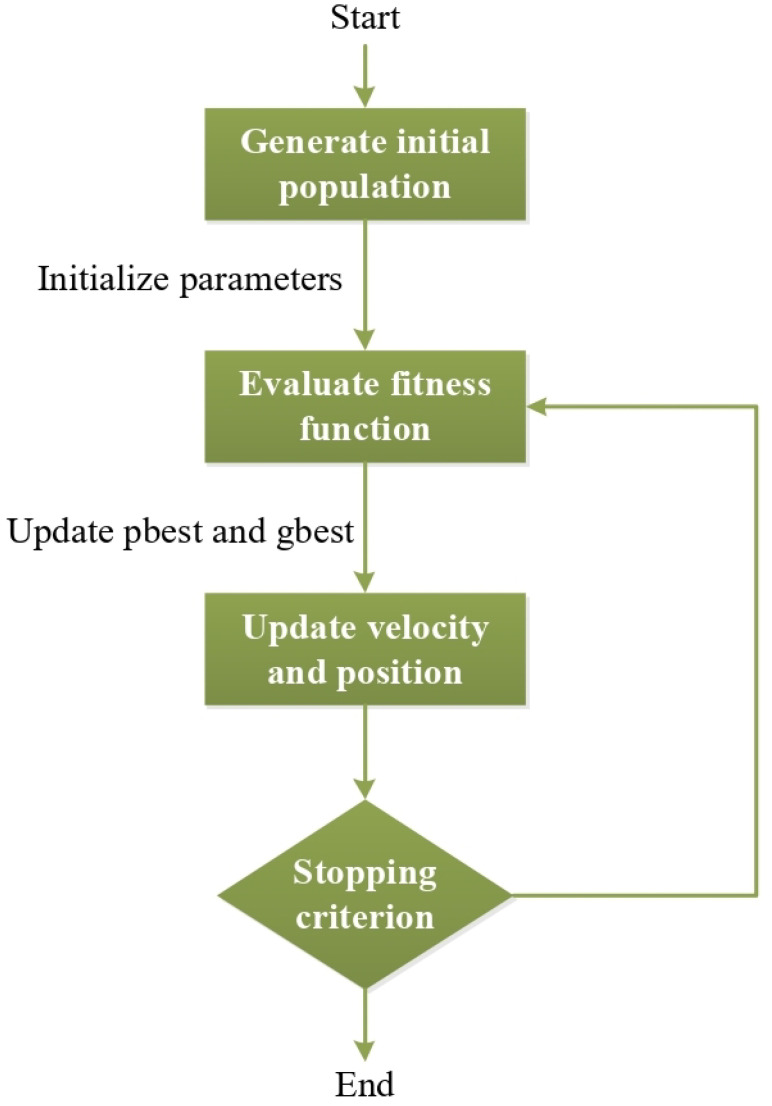
PSO flow diagram.

The Particle Swarm Optimization (PSO) algorithm is a widely used optimization technique grounded in swarm intelligence principles [14]. It is widely applied in the literature for addressing various optimization problems. PSO operates by facilitating social information sharing within the swarm, enabling individuals’ positions to converge towards the best position within the swarm [15]. Figure 1 illustrates the flow diagram of the PSO algorithm:

In the flow diagram illustrated in Figure 1, the algorithm commences by randomly dispersing particles, each representing a potential solution for the problem, across the solution space. During this step, other parameters within the PSO are also established. Subsequently, the fitness value of all the particles in the swarm is computed, indicating the quality of solutions contained within each particle. Following this, the particle with the best position in the current iteration (pbest) is identified based on its fitness value. Then, the global best (gbest) is determined among the particles possessing the best fitness value in the current iteration. In the subsequent step, the velocities of the particles and their corresponding positions are updated based on this new velocity value, as outlined in Equations (1) and (2):

(1) $$V_{(i+1)j}=WV_{ij}+c_{1}rand_{1}\left[pbest_{i}-X_{ij}\right]+c_{2}rand_{2}\left[gbest_{i}-X_{ij}\right]$$

(2) $$X_{ij}\left(t+1\right)=X_{ij}\left(t\right)+V_{ij}\left(t+1\right)$$

where $V_{ij}$ represents the velocity of particle *j* at iteration *i*; $rand_{1}$ and $rand_{2}$ represent random values generated in the range [0–1]; $c_{1}$ represents the cognitive learning ability within the swarm, while $c_{2}$ represents the social learning ability within the swarm; $X_{ij}$ represents the position of particle *j* in iteration *i*; $pbest_{i}$ represents the best particle at iteration *i*; and $gbest_{i}$ represents the global best particle [16].

The algorithm iterates until the stopping criterion is satisfied. Upon meeting the stopping criterion, the pbest and gbest values are updated, and the best particle suitable for the problem is determined.

### 3.2. Stacked AutoEncoders

Stacked AutoEncoders (SAE) is a type of stacked unsupervised Neural Network comprising AutoEncoder (AE) Neural Networks. An AE Neural Network consists of two main components: an encoder and a decoder. The encoder is responsible for transforming input data into a compressed representation, known as features, while the decoder reconstructs the original input data from these features.

**Figure 2:**
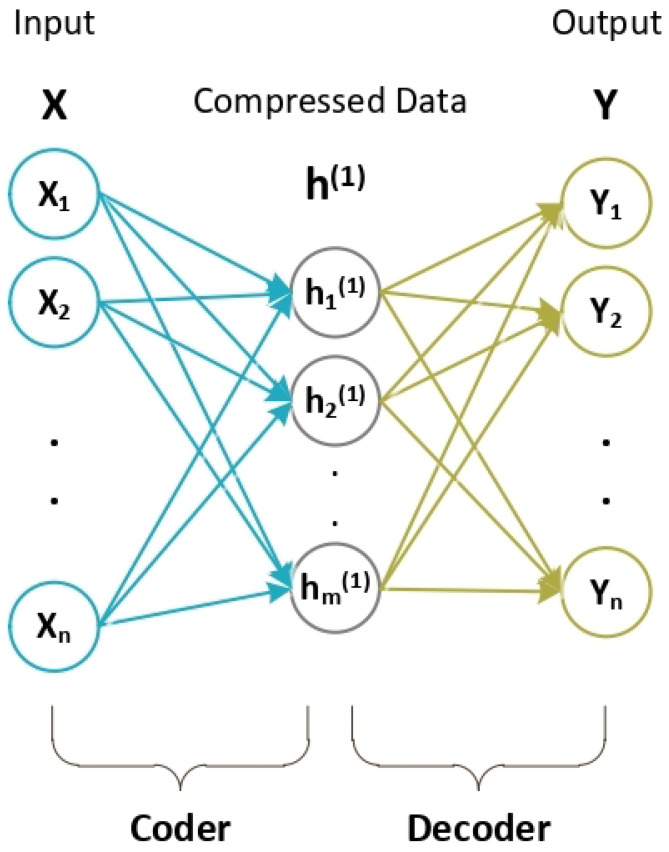
AutoEncoder structure.

Figure 2 illustrates the basic structure of an AE, which consists of three layers. In an AE, the output of each hidden layer serves as the input data for the subsequent hidden layer. An AE comprises two main components: the encoder and the decoder. AEs facilitate data size reduction by extracting features through the encoder component.

**Figure 3:**
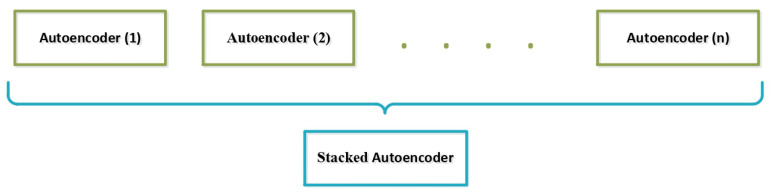
Stacked AutoEncoders structure.

Multiple AutoEncoders (AEs) are combined to form a Stacked AutoEncoders (SAE) Neural Network. Figure 3 illustrates the structure of the SAE.

### 3.3. The Softmax Classifier

The Softmax Classifier is a Machine Learning method commonly employed in multi-layer Artificial Neural Network (ANN) architectures, particularly in classification tasks [17,18]. The mathematical representation of the softmax function is depicted in Equation (3) [19]:

(3) $$\sigma {\left(z\right)}_{j}=\frac{e^{z_{j}}}{\sum_{k=1}^{K}e^{z_{k}}}$$

Here, $\sigma {\left(z\right)}_{j}$ represents the softmax output calculated for class *j*; *e* represents the Euler number; $z_{j}$ represents the element of the input vector with index *j*; *K* represents the number of classes.

### 3.4. Evaluation Metrics

**Figure 4:**
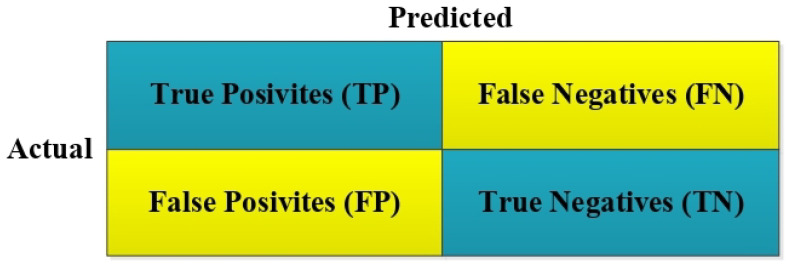
Confusion matrix for a two-class classifier.

The Accuracy, Sensitivity, Precision, and F-score metrics used in the literature to measure the performance of classification problems were applied in estimating postoperative patient survival [20,21]. In order to calculate these performance measurement metrics, the confusion matrix of the classification process had first be determined [22,23]. A confusion matrix is a tabular layout that allows visualizing the performance of an algorithm [24]. An example confusion matrix is shown in Figure 4:

TP in Figure 4 represents the True Positive value. TN represents True Negative. FP represents False Positive. FN represents False Negative. The mathematical formula of Accuracy, Sensitivity, Precision, and F-score metrics over the complexity matrix is shown in Equations (4)–(7) [25]:

(4) $$Accuracy\left(A\right)=\frac{TP+TN}{TP+TN+FN+FP}$$

(5) $$Sensitivity\left(S\right)=\frac{TP}{FN+TP}$$

(6) $$Precision\left(P\right)=\frac{TP}{TP+FP}$$

(7) $$F\text{-}Score=\frac{2\times P\times S}{P+S}$$

Sensitivity indicates the ratio of positively classified data to the actual positive data. Precision represents the ratio of correctly classified data. F-Score provides the harmonic mean of Precision and Sensitivity values, providing a complete picture of accuracy. The Accuracy value represents the accuracy itself.

### 3.5. Other Machine Learning Methods Used in the Study

Apart from the proposed architecture, the other Machine Learning methods applied on the same dataset are presented in Table 1.

The Machine Learning methods presented in Table 1 are used in many classification problems [32,33,34].

## 4. Datasets

In this section, detailed information about the various datasets utilized in the study will be provided.

### 4.1. Echocardiogram Dataset

One of the datasets used in the study is the Echocardiogram dataset. This dataset was obtained from UCI, which is publicly available [35]. The primary challenge addressed by the researchers is to predict whether a patient will survive for at least one year based on other variables. Nine meaningful features were considered in the dataset. The features included in the dataset are shown in Table 2.

**Figure 5:**
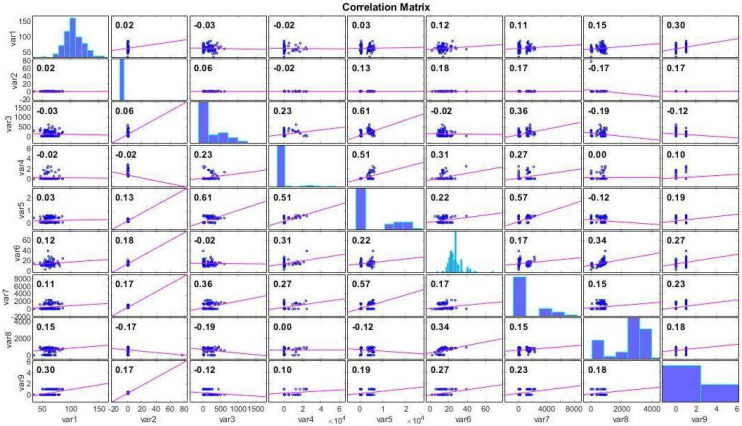
Echocardiogram dataset correlation chart.

There are a total of 132 data in the dataset whose characteristics are given in Table 2. Since no information could be obtained about 1 tansi among these data, this datum was excluded from the dataset. Experimental studies were conducted on these 131 data and the dataset was divided into two parts, 70% training and 30% testing. The missing information in the dataset made the predictability of the dataset difficult. The correlation of the dataset features with each other is shown in Figure 5:

In Figure 5, the correlation between each feature in the dataset and other features is depicted. For instance, a positive correlation is observed between the Age-at-heart-attack feature (var1) and the Pericardial-effusion (var2) feature, while a negative correlation exists between the Pericardial-effusion (var2) feature and Epss (var4).

### 4.2. Haberman’s Survival Dataset

In the study, the Haberman’s Survival dataset available at UCI was used for postoperative patient survival prediction [36]. The dataset characteristics are shown in Table 3.

In the dataset, whose characteristics are presented in Table 1, there are a total of 306 data, 225 of which are indicated as ’patient survived for 5 years or more’ and 81 of which are indicated as ’patient died within 5 years’. The dataset was divided into two different groups for the training and testing phases: 70% of the dataset was used for training and 30% for testing.

**Figure 6:**
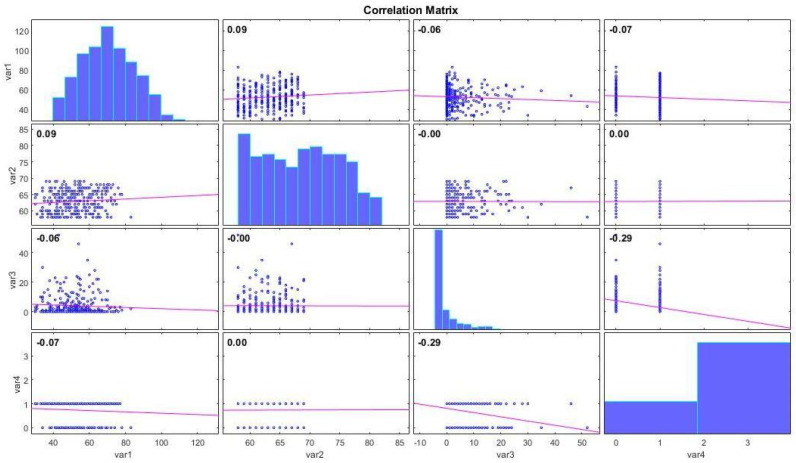
Haberman’s Survival dataset correlation chart.

The correlation of the dataset features with each other is shown in Figure 6:

In Figure 6, the correlation between each feature in the dataset and other features is illustrated. For example, a positive correlation is observed between the ’Age of the patient at the time of operation’ feature (var1) and ’Patient’s year of operation’ (var2), while a negative correlation is evident between the ’Number of positive axillary nodes detected’ (var3) feature and ’Survival status’ (var4).

## 5. Proposed Model

**Figure 7:**
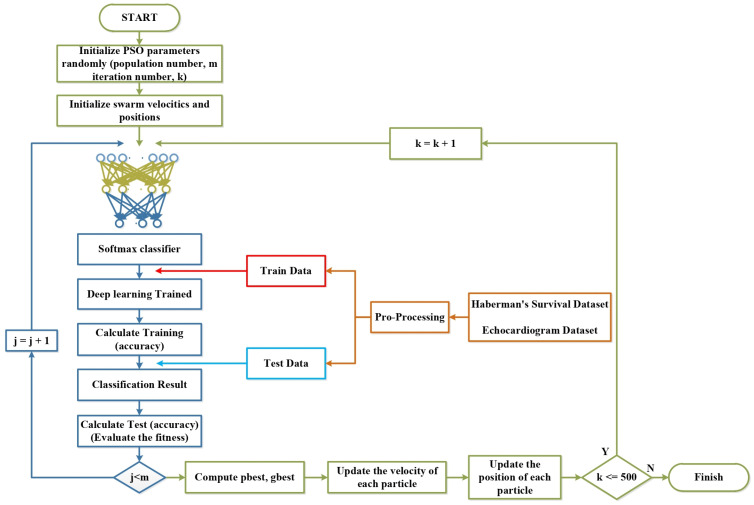
The proposed SAE–Softmax Classifier–PSO hybrid architecture.

An optimization-based hybrid architecture for predicting survival on datasets was created. The architecture is shown in Figure 7.

In the architecture presented in Figure 7, a Softmax classifier is connected to the Stacked AutoEncoders output for survival time estimation. Considering the resulting architecture and the layers used in the architecture, there were too many hyperparameters. An optimization algorithm was used to determine these hyperparameters in the hybrid architecture created with PSO–SAE–Softmax Classifier and the parameters of the architecture to produce the best result. PSO was used in this study, due to its minimum parameter requirement, success in solving multidimensional problems, and ease of implementation. The optimization-based hybrid architecture was implemented on the MATLAB platform for coding flexibility and ease of working with multidimensional matrices [37]. The figures and graphs in the following sections of the study were plotted in the MATLAB software (MATLAB R2021a) environment. The parameters to be optimized in the architecture are shown in Table 4.

The hyperparameters in Table 4 directly affect the success performance of the network. In such a problem, with a nine-dimensional solution space, the most successful architecture and hyperparameter values can only be determined by optimization algorithms [16,38]. One of the most significant features of the hybrid model is that each particle within the model possesses a unique architecture and distinct hyperparameters from the others. This hybrid model not only optimizes the architecture’s structure but also fine-tunes the hyperparameters specific to each particle’s architecture, thereby aiming to achieve optimal results.

As a first step in the proposed Deep Learning architecture, the PSO parameters that will optimize the structure and hyperparameters of the architecture were determined, as shown in Table 5, as a result of the experimental studies.

Given the expansive solution space and the expectation for the optimization algorithm to fine-tune both hyperparameters and architecture, a substantial number of iterations was maintained. Each member of the population, as outlined in Table 5, encompassed all the hyperparameters detailed in Table 4.

In the subsequent step, a number of particles equal to the population specified in Table 5 was randomly distributed within the solution space. The NSAE value contained within each particle, as outlined in Table 4, was randomly assigned in accordance with the constraint function delineated in Equation (8):

(8) $$P_{i}NSAE_{j}\left(n\right)=\left\{\begin{array}{cc} 1 & n<1\\ n & 1\leq n\leq 6\\ 6 & n>6 \end{array}\right.$$

where $P_{i}NSAE_{j}\left(n\right)$ represents the NSAE value of particle *i* at iteration *j*.

The NHED values depicted in Table 4 were randomly assigned based on the constraint function described in Equation (9):

(9) $$P_{i}NHED_{j}\left(n\right)=\left\{\begin{array}{cc} 10 & n<10\\ n & 10\leq n\leq 250\\ 250 & n>250 \end{array}\right.$$

where $P_{i}NHED_{j}\left(n\right)$ represents the NHED value of particle *i* at iteration *j*.

The NEAF values depicted in Table 4 were randomly assigned based on the constraint function described in Equation (10):

(10) $$P_{i}NEAF_{j}\left(n\right)=\left\{\begin{array}{cc} 1 & n<1\\ n & 1\leq n\leq 2\\ 2 & n>2 \end{array}\right.$$

where $P_{i}NEAF_{j}\left(n\right)$ represents the NEAF value of particle *i* at iteration *j*. The activation functions corresponding to the randomly determined NEAF value are listed in Table 6.

The NDAF values depicted in Table 4 were randomly assigned based on the constraint function described in Equation (11):

(11) $$P_{i}NDAF_{j}\left(n\right)=\left\{\begin{array}{cc} 1 & n<1\\ n & 1\leq n\leq 3\\ 3 & n>3 \end{array}\right.$$

where $P_{i}NDAF_{j}\left(n\right)$ represents the NDAF value of particle *i* at iteration *j*. The activation functions corresponding to the randomly determined NDAF value are listed in Table 7.

The AEL2 values depicted in Table 4 were randomly assigned based on the constraint function described in Equation (12):

(12) $$P_{i}AEL2_{j}\left(n\right)=\left\{\begin{array}{cc} 0.001 & n<0.001\\ n & 0.001\leq n\leq 0.01\\ 0.01 & n>0.01 \end{array}\right.$$

where $P_{i}AEL2_{j}\left(n\right)$ represents the AEL2 value of particle *i* at iteration *j*.

The AESR values depicted in Table 4 were randomly assigned based on the constraint function described in Equation (13):

(13) $$P_{i}AESR_{j}\left(n\right)=\left\{\begin{array}{cc} 1 & n<1\\ n & 1\leq n\leq 5\\ 5 & n>5 \end{array}\right.$$

where $P_{i}AESR_{j}\left(n\right)$ represents the AESR value of particle *i* at iteration *j*.

The AESP values depicted in Table 4 were randomly assigned based on the constraint function described in Equation (14):

(14) $$P_{i}AESP_{j}\left(n\right)=\left\{\begin{array}{cc} 0 & n<0\\ n & 0\leq n\leq 1\\ 1 & n>1 \end{array}\right.$$

where $P_{i}AESP_{j}\left(n\right)$ represents the AESP value of particle *i* at iteration *j*.

The AELD values depicted in Table 4 were randomly assigned based on the constraint function described in Equation (15):

(15) $$P_{i}AELD_{j}\left(n\right)=\left\{\begin{array}{cc} 1 & n<1\\ n & 1\leq n\leq 2\\ 2 & n>2 \end{array}\right.$$

where $P_{i}AELD_{j}\left(n\right)$ represents the AELD value of particle *i* at iteration *j*. The values corresponding to the randomly determined AELD value are shown in Table 8.

After randomly distributing the particles across the solution space, the fitness values of each particle were computed according to Equation (16):

(16) $$FP_{ij}=Test\left(Accuracy{\left(SAESoft\right)}_{ij}\right)$$

where $FP_{ij}$ denotes the fitness value of particle *i* at iteration *j*, and where ${\left(SAESoft\right)}_{ij}$ represents the architecture of particle *i* at iteration *j*. Equation (16) governed the calculation of the fitness value for each particle. Based on the computed fitness value, pbest (particle best) and gbest (global best) were determined.

In the subsequent step of the architecture, the velocity values of each particle were updated following Equation (1). In this process, the new velocities of each particle were determined based on the constraint functions utilized in the population formation. After adjusting the velocities, the position of each particle was updated according to the mathematical expression provided in Equation (2).

The hybrid architecture was separately executed for both datasets, following the number of iterations outlined in Table 4. In the final step, the fitness values of each particle within the hybrid architecture were computed separately for both datasets, using Equation (16), and the global best values were determined based on the experimental studies conducted with both datasets. The architectural parameters and hyperparameters associated with the particle exhibiting the best gbest value from the experimental studies for the Haberman’s Survival dataset are presented in Table 9.

**Figure 8:**
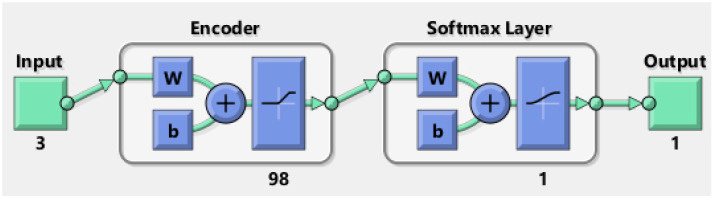
Survival prediction model after breast cancer surgery.

The hybrid architecture obtained with the parameters shown in Table 9 as a result of the experimental studies for Haberman’s Survival dataset is shown in Figure 8.

As depicted in Figure 8, the input values traversed through a 98-layer Encoder section before being connected to the Softmax Classifier. Here, they underwent the classification process, resulting in the production of output.

The architectural parameters and hyperparameters associated with the particle exhibiting the best gbest value from the experimental studies for the Echocardiogram dataset are presented in Table 10.

**Figure 9:**
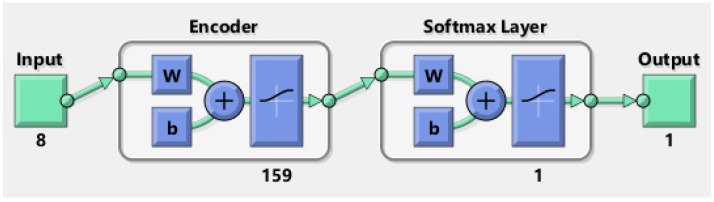
Survival prediction model after heart attack.

The hybrid architecture obtained with the parameters shown in Table 10 as a result of the experimental studies for the Echocardiogram dataset is shown in Figure 9.

As depicted in Figure 9, the input values traversed through a 159-layer Encoder section before being connected to the Softmax Classifier. Here, they underwent the classification process, resulting in the production of output.

**Figure 10:**
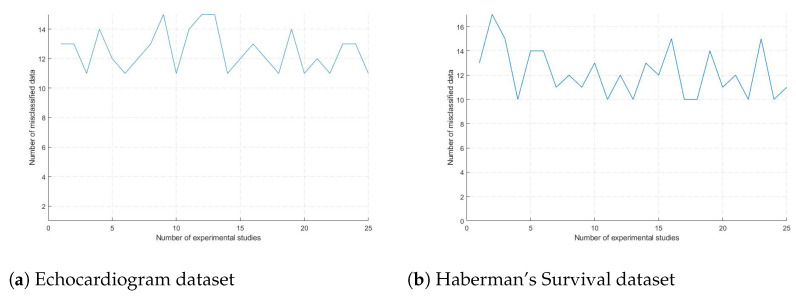
Hybrid architecture performance in experimental studies.

Acknowledging the potential for the PSO to become trapped in local optima, the architecture depicted in Figure 7 was executed 25 times for each dataset. Models were generated using the optimal parameters obtained from these runs. The performance of the architecture across each experimental run for the datasets is depicted in Figure 10.

The results from the experimental investigations illustrated in Figure 10 were analyzed by assessing the model’s minimum misclassification performance in each experimental trial.

Furthermore, the computational complexity of both the hybrid architecture proposed in this study and the other Machine Learning methods utilized are displayed in Table 11.

Upon reviewing Table 11, it becomes evident that the proposed method exhibits remarkable complexity performance.

## 6. The Experimental Results and Discussion

### 6.1. Survival Prediction Model after Heart Attack

**Figure 11:**
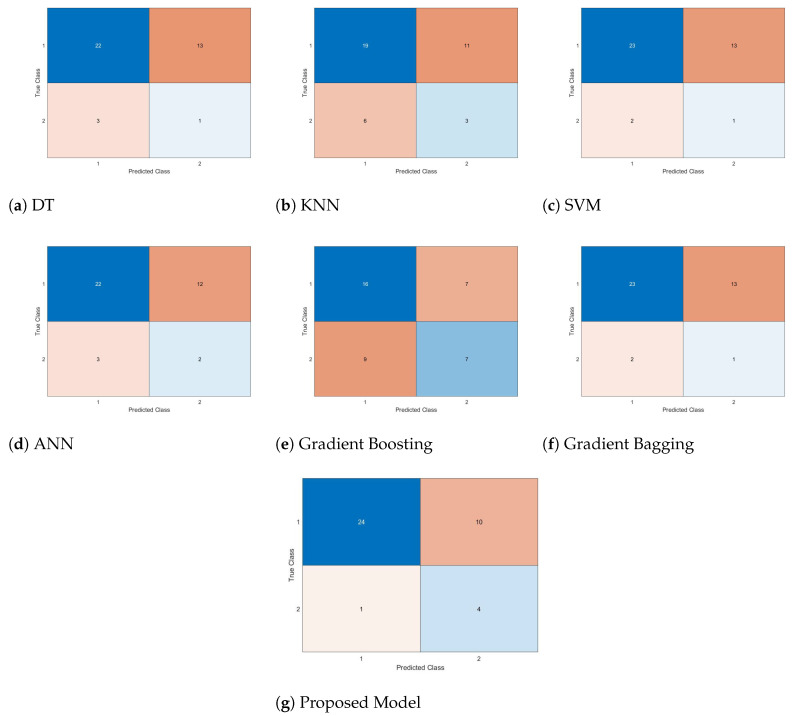
Confusion matrices of models for predicting survival after heart attack.

In this study, in addition to the architecture outlined for the survival datasets, commonly utilized methods such as Decision Trees (DT), K-Nearest Neighbors (KNN), Support Vector Machines (SVMs), Neural Networks (NN), Gradient Boosting, and Gradient Bagging were also employed. All methodologies utilized in this study underwent training with allocated training datasets and subsequent testing with independent test datasets. The experimental findings, conducted with the Chocardiogram dataset, are presented through the confusion matrices of the models evaluated with the test dataset, as illustrated in Figure 11.

When examining the confusion matrices obtained from the methods illustrated in Figure 11, it becomes apparent that KNN, exhibiting the highest number of incorrect groupings among its predictions, is the least effective predictor of survival times after a heart attack. Conversely, the proposed hybrid model emerges as the most accurate estimator. Utilizing the confusion matrices provided in Figure 11 and the evaluation metrics summarized in Equations (4) to (7), the performance percentages of the models are calculated and presented in Table 12.

Based on the metric values summarized in Table 12, it is evident that the proposed hybrid architecture attained the highest performance metrics and accuracy rate. Upon evaluating these results, it becomes apparent that the hybrid model, optimized with the optimization-based hybrid architecture, outperforms other Machine Learning methods.

### 6.2. Survival Prediction Model after Breast Cancer Surgery

**Figure 12:**
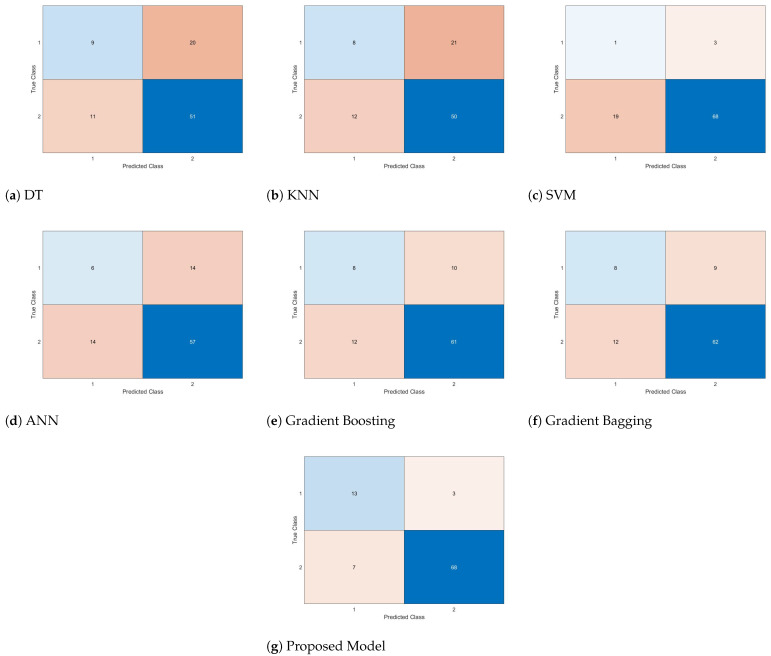
Confusion matrices of models for predicting survival after breast cancer surgery.

The confusion matrices resulting from experimental studies on the test dataset for survival prediction after breast cancer surgery, comparing the hybrid architecture with other methods, are depicted in Figure 12.

When the confusion matrices obtained from the methods shown in Figure 12 are analyzed, it is seen that Decision Trees (DT), which has the highest number of incorrect groupings among its predictions, is the weakest predictor of survival times after breast cancer surgery, while the proposed hybrid model emerges as the most accurate predictor. Based on the confusion matrices provided in Figure 12 and the evaluation metrics outlined in Equations (4)–(7), the performances of the models are computed and presented in Table 13.

Based on the metric values outlined in Table 13, although the SVM exhibits the highest precision value, the proposed hybrid architecture achieved the highest accuracy rate. Evaluating these results, it becomes evident that the hybrid model optimized with the optimization-based hybrid architecture attains more successful outcomes compared to other Machine Learning methods.

The comparative results of the outcomes obtained using the proposed optimization-based hybrid architecture with those from studies conducted in the literature employing the same dataset are presented in Table 14.

As depicted in Table 14, the proposed hybrid Deep Learning architecture achieved superior results compared to the other studies in the literature employing the same dataset.

## 7. Conclusions

Survival prediction models that achieve high accuracy play a critical role in patient treatments. By more accurately estimating patients’ survival probabilities, these models help optimize treatment processes, offering more effective and personalized treatment strategies to patients, thereby improving health outcomes. Additionally, they provide healthcare professionals with the opportunity to manage patient care better by distributing resources more efficiently and providing early warnings to identify high-risk situations. Therefore, survival prediction models that achieve high accuracy are essential tools in clinical decision-making processes, enhancing the quality of healthcare services. In this study, we present an optimization-based hybrid method that integrates SAE, PSO, and the Softmax Classifier to predict the survival of patients undergoing breast cancer surgery and those experiencing a heart attack. The architecture derived from our proposed hybrid method was applied to the Haberman’s Survival and Echocardiogram datasets from UCI. In addition to our proposed architecture, various methods, including DT, SVMs, KNN, ANN, Gradient Boosting, and Gradient Bagging were also applied on the same datasets. Our proposed architecture achieved 72% and 89% accuracy rates on the datasets used in the study, respectively, and provided superior results compared to the other methods. Furthermore, the outcomes obtained with our proposed architecture outperformed previous studies in the literature on the Haberman’s Survival dataset. With the proposed optimization-based hybrid architecture:

- Various Deep Learning architectures based on optimization have been developed to predict survival after breast cancer surgery and the survival of patients following a heart attack.
- Utilizing Particle Swarm Optimization (PSO), both architecture parameters and hyperparameters within the architecture are optimized.
- Through leveraging features from the Haberman’s Survival dataset, a model exhibiting superior performance compared to the existing literature studies has been achieved.
- The optimized architecture, facilitated by the developed Deep Learning framework, offers access to both features and hyperparameters.
- The hybrid Deep Learning Network created is not only tailored to this particular problem but can also be seamlessly applied to various other problems.
- The architecture obtained through the hybrid Deep Learning Network stands as a viable alternative method for integration into health decision support systems.

In future studies, the findings can pave the way for developing applications tailored for smart devices. 

## Figures and Tables

**Table 1:** Machine Learning methods used in the study.

Methods	Information
Decision Trees (DT)	It is a tree-like model that obtains results by dividing and classifying the dataset with successive decisions [26].
K-Nearest Neighbors (KNN)	It is a method that predicts the class or value of a data point based on the class of the K data points closest to it [27].
Support Vector Machines (SVMs)	It is a method that aims to find an optimal hyperplane to best classify or predict data points [28].
Artificial Neural Networks (ANNs)	Artificial Neural Networks are a mathematical representation of nerve cells found in humans [29].
Gradient Boosting	Gradient Boosting is a widely used Machine Learning technique that has proven highly effective in batch learning [30].
Gradient Bagging	Gradient Bagging is a Machine Learning method that shows high performance in classification problems [31].

**Table 2:** Echocardiogram dataset properties and descriptions.

Features	Variable Information	Data Type
Age-at-heart-attack	Numerical	Input
Pericardial-effusion	Binary	Input
Fractional-shortening	Numerical	Input
Epss	Numerical	Input
Lvdd	Numerical	Input
Wall-motion-score	Numerical	Input
Wall-motion-index	Numerical	Input
Multi	Numerical	Input
Still-alive	Class attribute 0—the patient is dead at end of survival period 1—the patient is still alive	Output

**Table 3:** Dataset properties and descriptions.

Features	Variable Information	Data Type
Age of patient at time of operation	Numerical	Input
Patient’s year of operation	Numerical	Input
Number of positive axillary nodes detected	Numerical	Input
Survival status	Class attribute 0—the patient survived 5 years or longer 1—the patient died within 5 years Output	Output

**Table 4:** Parameters used in the architecture and to be optimized.

Parameters to Optimize	Parameter Feature	Representation in Architecture
Number of AutoEncoders in the Stacked AutoEncoder architecture	Hyperparameter in Hybrid Architecture structure	NSAE
Number of hidden layers in the encoder layer in each AutoEncoder in the architecture	Hyperparameter in the Stacked AutoEncoders structure	NHED
Number of hidden layers in the decoder layer in each AutoEncoder in the architecture	Hyperparameter in the Stacked AutoEncoders structure	NHED
Activation functions used in the hidden layers in the encoder section of each AutoEncoder structure	Hyperparameter in the Stacked AutoEncoders structure	NEAF
Activation functions used in the hidden layers in the decoder section of each AutoEncoder structure	Hyperparameter in the Stacked AutoEncoders structure	NDAF
L2WeightRegularization coefficient used in the AutoEncoder structure	Hyperparameter in the Stacked AutoEncoders structure	AEL2
SparsityRegularization coefficient used in the AutoEncoder structure	Hyperparameter in the Stacked AutoEncoders structure	AESR
SparsityProportion coefficient used in the AutoEncoder structure	Hyperparameter in the Stacked AutoEncoders structure	AESP
Lscaledata coefficient used in the AutoEncoder structure	Hyperparameter in the Stacked AutoEncoders structure	AELD

**Table 5:** Parameters used in PSO.

PSO Parameters	Values
Number of particles	40
Solution Space	9
Iteration Number	500

**Table 6:** Activation functions corresponding to the NEAF value.

NEAF Values	Activation Function
1	logsig
2	satlin

**Table 7:** Activation functions corresponding to the NDAF value.

NDAF Values	Activation Function
1	logsig
2	satlin
3	purelin

**Table 8:** Values corresponding to the AELD value.

AELD Values	Values
1	true
2	false

**Table 9:** Parameters and their values in the gbest particle for the Haberman’s Survival dataset.

Parameters	Values
NSAE	1
NHED	98
NEAF	2 (satlin)
NDAF	2 (satlin)
AEL2	0.0063
AESR	3.96
AESP	0.16
AELD	2 (false)

**Table 10:** Parameters and their values in the gbest particle for the Echocardiogram dataset.

Parameters	Values
NSAE	1
NHED	159
NEAF	1 (logsig)
NDAF	2 (satlin)
AEL2	0.0038
AESR	4.64
AESP	0.56
AELD	1 (true)

**Table 11:** Computational complexities of the methods used in the study.

Methods	Computational Complexity	Parameters
DT	O(H)	H: height of tree
KNN	O(MLog(k)NLog(N))	M: number of features
SVM	O(N2)	k: number of neighbors
ANN	O(hNM)	N: number of observations
Gradient Boosting	O(d N logn d)	d: number of trees
Gradient Bagging	O(MNlog(N))	h: number of hidden neurons
Proposed Model	O(iNK+iMhN)	K: sum number of hidden neurons of AE
		i: number of iterations

**Table 12:** Performance of methods used in survival after heart attack.

Models	Precision	Recall	F-Score	Accuracy
DT	62.28	88	73.33	59
KNN	63.33	76	69.09	56
SVM	63.89	92	75.40	62
ANN	64.70	88	74.58	62
Gradient Boosting	69.56	64	66.67	59
Gradient Bagging	63.89	92	75.4	62
Proposed architecture	**70.59**	**96**	**81.35**	**72**

**Table 13:** Performance of methods used in survival after breast cancer surgery.

Models	Precision	Recall	F-Score	Accuracy
DT	31.10	45	36.73	66
KNN	27.59	40	32.65	64
SVM	25	50	9.33	76
ANN	30	30	30	69
Gradient Boostng	44.44	40	42.10	76
Gradient Bagging	47.06	40	43.24	77
Proposed architecture	**81.25**	**65**	**72.22**	**89**

**Table 14:** Literature comparison with the proposed method for the Haberman’s Survival dataset.

Authors	Year	Method	Accuracy (%)	F-Score
Bataineh et al. [10]	2022	Clonal Selection Algorithm + MLP	76.10	
Remya Ajai et al. [13]	2023	Heterogeneous Neurochaos Learning Architecture	77.42	0.72
Sethi et al. [39]	2023	Neurochaos Learning features+Random Forest		0.61
Melin at al. [40]	2024	IT3FIS-GA	77.05	
Proposed method	2024	SAE-SOFTMAX-PSO	89	0.72

## Data Availability

This study used the Haberman’s Survival dataset and the Echocardiogram dataset, both of which are publicly available at the UC Irvine Machine Learning Repository. (https://archive.ics.uci.edu/dataset/43/haberman+s+survival, https://archive.ics.uci.edu/dataset/38/echocardiogram), accessed on 20 April 2024.
